# Psychological trauma and the genetic overlap between posttraumatic stress disorder and major depressive disorder

**DOI:** 10.1017/S0033291721000830

**Published:** 2021-06-04

**Authors:** Jessica Mundy, Christopher Hübel, Joel Gelernter, Daniel Levey, Robin M. Murray, Megan Skelton, Murray B. Stein, The Million Veteran Program, Post Traumatic Stress Disorder Working Group of the Psychiatric Genomics Consortium, Evangelos Vassos, Gerome Breen, Jonathan R. I. Coleman

**Affiliations:** 1 Social, Genetic and Developmental Psychiatry Centre; Institute of Psychiatry, Psychology & Neuroscience, King’s College London, London, UK; 2 UK National Institute for Health Research (NIHR) Biomedical Research Centre, South London and Maudsley National Health Service (NHS) Trust, London, UK; 3 Department of Medical Epidemiology and Biostatistics, Karolinska Institutet, Stockholm, Sweden; 4 Division of Human Genetics, Department of Psychiatry, Yale University School of Medicine, New Haven, Connecticut, USA; 5 Department of Psychiatry, Veterans Affairs Connecticut Healthcare Center, West Haven, Connecticut, USA; 6 Departments of Genetics and Neuroscience, Yale University School of Medicine, New Haven, Connecticut, USA; 7 Institute of Psychiatry, Psychology & Neuroscience, King’s College London, London, UK; 8 Psychiatry Service, VA San Diego Healthcare System, San Diego, California, USA; 9 Departments of Psychiatry and Family Medicine & Public Health, University of California San Diego, La Jolla, California, USA

**Keywords:** Posttraumatic stress disorder, major depressive disorder, psychological trauma, genetics, genetic correlations, polygenic risk scores

## Abstract

**Background.:**

Posttraumatic stress disorder (PTSD) and major depressive disorder (MDD) are commonly reported co-occurring mental health consequences of psychological trauma exposure. The disorders have high genetic overlap. Trauma is a complex phenotype but research suggests that trauma sensitivity has a heritable basis. We investigated whether sensitivity to trauma in those with MDD reflects a similar genetic component in those with PTSD.

**Methods.:**

Genetic correlations between PTSD and MDD in individuals reporting trauma and MDD in individuals not reporting trauma were estimated, as well as with recurrent MDD and single-episode MDD, using genome-wide association study (GWAS) summary statistics. Genetic correlations were replicated using PTSD data from the Psychiatric Genomics Consortium and the Million Veteran Program. Polygenic risk scores were generated in UK Biobank participants who met the criteria for lifetime MDD (*N* = 29 471). We investigated whether genetic loading for PTSD was associated with reporting trauma in these individuals.

**Results.:**

Genetic loading for PTSD was significantly associated with reporting trauma in individuals with MDD [OR 1.04 (95% CI 1.01–1.07), Empirical-*p* = 0.02]. PTSD was significantly more genetically correlated with recurrent MDD than with MDD in individuals not reporting trauma (*r*_g_ differences = ~0.2, *p <* 0.008). Participants who had experienced recurrent MDD reported significantly higher rates of trauma than participants who had experienced single-episode MDD (χ^2^ > 166, *p* < 0.001)

**Conclusions.:**

Our findings point towards the existence of genetic variants associated with trauma sensitivity that might be shared between PTSD and MDD, although replication with better powered GWAS is needed. Our findings corroborate previous research highlighting trauma exposure as a key risk factor for recurrent MDD.

## Introduction

Symptoms of posttraumatic stress disorder (PTSD) and major depressive disorder (MDD) are the most commonly described co-occurring problems following exposure to psychological trauma (Ben Barnes, Hayes, Contractor, Nash, & Litz, 2018). Across epidemiological samples, approximately 50% of individuals with PTSD have a comorbid diagnosis of MDD (Breslau, Davis, Peterson, & Schultz, 1997; Kessler, Sonnega, Bromet, Hughes, & Nelson, 1995; Rytwinski, Scur, Feeny, & Youngstrom, 2013). Similar or occasionally higher estimates are observed in primary care settings (Alim et al., 2006; Stein, McQuaid, Pedrelli, Lenox, & McCahill, 2000). Previously, high comorbidity rates were attributed to the classification of shared symptoms into the two diagnostic categories (Flory & Yehuda, 2015), such as negative mood, sleep disturbances, irritability and concentration difficulties (American Psychiatric Association, 2013). However, several studies demonstrate that comorbidity rates do not diminish after excluding shared symptoms from clinical diagnoses (Elhai et al., 2011; Grubaugh, Long, Elhai, Frueh, & Magruder, 2010), suggesting that symptom overlap does adequately explain comorbidity. An alternative explanation might be the genetic overlap between the disorders (Sartor et al., 2012). Twin studies have previously indicated that PTSD shares genetic influences with MDD (*r* = 0.77) and related conditions (Koenen et al., 2008; Wolf et al., 2010). More recently, methods based on genome-wide association studies (GWAS) have been used to explore genetic correlations (*r*_g_), a quantitative measure of the genetic relationship between two polygenic traits (van Rheenen, Peyrot, Schork, Lee, & Wray, 2019). Research from the Psychiatric Genomics Consortium (PGC) reported strong, positive genetic correlations of PTSD with depressive symptoms (*r*_g_ = 0.80) and with MDD (*r*_g_ = 0.62) (Nievergelt et al., 2019), thus supporting results from twin studies.

As well as shared genetics, a potential factor involved in PTSD-MDD comorbidity is exposure to trauma. There is a complex relationship between trauma exposure and mental health sequelae. Exposure to trauma is common (Breslau, Davis, Andreski, & Peterson, 1991; Kessler et al., 1995). In total, 50–90% of people will experience a traumatic event in their lifetime but only 8–12% will go on to develop PTSD (Shah, Shah, & Links, 2012), suggesting that certain individuals are at a greater risk of developing PTSD than others following exposure (Auxéméry, 2012; Duncan et al., 2018b; Nievergelt et al., 2019). Similarly, stressful and traumatic events are significant risk factors for MDD (Horesh, Klomek, & Apter, 2008; Hovens, 2015 ; Shapero et al., 2014), but the majority of people who are exposed do not develop the disorder (Kessler, 1997). Therefore, similar to PTSD, the effects of these events on the risk of developing MDD may be moderated by individual liability or sensitivity to trauma.

Reporting adverse or traumatic life events is heritable (Dalvie et al., 2020; Jay Schulz-Heik et al., 2009; Plomin, Lichtenstein, Pedersen, McClearn, & Nesselroade, 1990; Power et al., 2013). However, this alone does not provide direct evidence for a genetic basis for trauma sensitivity. There are a number of additional factors that could contribute to trauma’s overall heritability, including whether or not the event is controllable and whether the individual plays an active or passive role in the event (Kendler, Karkowski, & Prescott, 1999; Plomin et al., 1990). Heritable personality traits are also important since these influence both the likelihood of exposure and willingness to report it (Sartor et al., 2012). We emphasise that the presence of these characteristics should not be interpreted as placing any blame on individuals who have experienced trauma. These heritable characteristics are difficult to disentangle from genetic influences on trauma sensitivity. However, studying mental health across individuals who have experienced trauma offers the opportunity to assess trauma sensitivity more specifically. Furthermore, with information on both life events and mental health in genotyped individuals, the extent that gene–environment interaction influences the development of psychopathology (such as the internalising symptoms in PTSD and MDD) can be investigated.

### Aims

The heritability of PTSD (Duncan et al., 2018b; Nievergelt et al., 2019; Stein et al., 2021), which by definition requires trauma exposure, indicates that variance in sensitivity to such events may be partially genetically influenced and interacts with environmental factors to influence individual risk for developing symptoms. Trauma is a key risk factor for MDD. Recent research found that in UK Biobank participants with MDD who reported traumatic life events, MDD had higher SNP-based heritability compared to MDD in participants not reporting trauma (24% *v.* 12% respectively), suggesting that trauma sensitivity also has a heritable basis in MDD (Coleman et al., 2020). PTSD and MDD co-occur often among trauma-exposed individuals and the disorders have substantial genetic overlap. In our study, we aimed to understand whether trauma sensitivity in those with MDD reflects a similar genetic component in those with PTSD.

We addressed our research question in two parts. First, we used GWAS summary statistics to calculate genetic correlations between PTSD and (1) MDD with reported trauma and (2) MDD without reported trauma. Given the evidence from clinical studies, we hypothesised that PTSD and MDD with reported trauma would demonstrate higher genetic overlap compared to PTSD and MDD without reported trauma. In the original GWAS of MDD with reported trauma, both cases and controls were trauma-exposed (Coleman et al., 2020). This means the summary statistics specifically capture genetic variants associated with MDD in individuals who report trauma. A higher genetic correlation with PTSD, which requires trauma exposure, would therefore reflect a shared genetically driven component associated with trauma sensitivity. We also used summary statistics to calculate genetic correlations between PTSD and (3) recurrent MDD and (4) single-episode MDD. Research has shown that the type, frequency and severity of traumatic events are associated with the frequency and severity of subsequent depressive episodes (Hovens, Giltay, Spinhoven, van Hemert, & Penninx, 2015; Nanni, Uher, & Danese, 2012; Otte et al., 2016), with childhood maltreatment being particularly associated with recurrence (Danese, 2020). Accordingly, our second hypothesis was that PTSD would show greater genetic overlap with recurrent MDD compared to single-episode MDD, under the assumption that trauma exposure is more likely to be reported by individuals with recurrent MDD. We tested the validity of this assumption in the participants.

Secondly, to address our research question further, we computed PTSD polygenic risk scores (PRS) in 29 471 UK Biobank participants who met criteria for lifetime MDD and tested their association with reporting trauma and MDD recurrence. Following the logic of our previous hypotheses, we expected that individuals with MDD with a higher genetic risk for PTSD would be more likely to report trauma and would be more likely to have experienced recurrent episodes than those with a lower genetic risk for PTSD.

## Methods

### Major depressive disorder

**Fig. 1.**
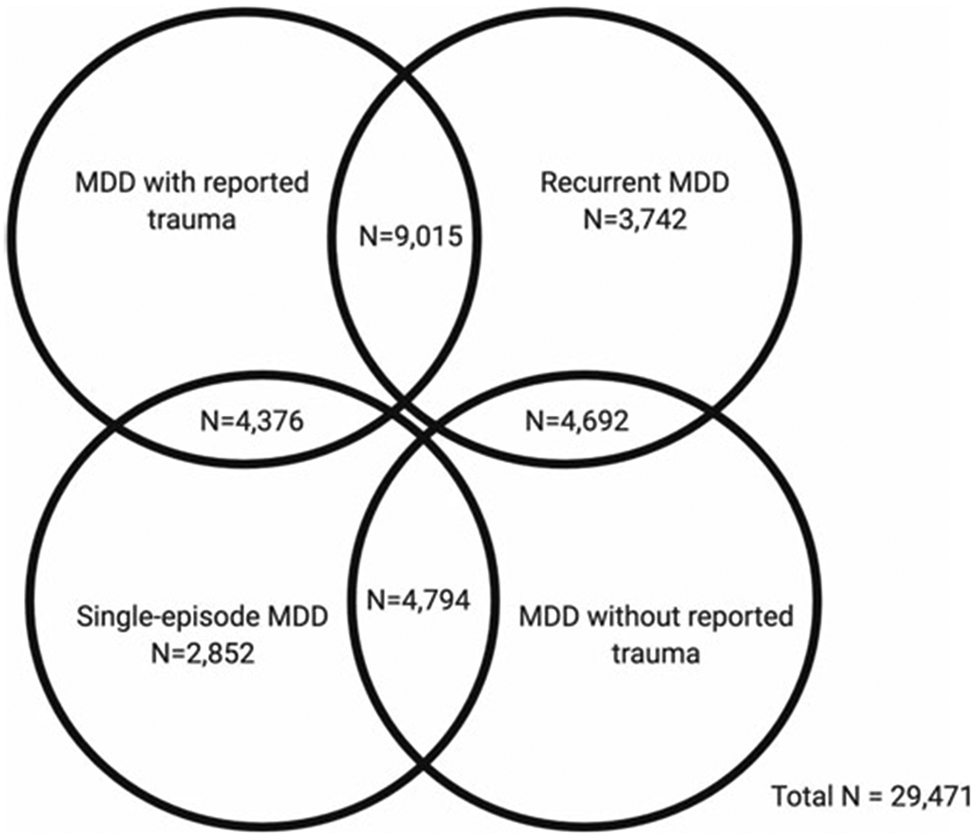
Venn diagram showing participant overlap between the four major depressive disorder (MDD) categories in UK Biobank Mental Health Questionnaire (MHQ) respondents who met criteria for lifetime MDD (*N* = 29 471). The MDD categories include MDD with reported trauma (*N* = 13 391), MDD without reported trauma (*N* = 9486), recurrent MDD (*N* = 17 449) and single-episode MDD (*N* = 12 022).

In the first part of our study, GWAS summary statistics for the four MDD categories were obtained from pre-existing studies (Table 1). In the second part, we analysed phenotypic and genomic data from 29 471 UK Biobank participants who met the criteria for lifetime MDD and had been included in the previous GWAS of the MDD categories. Four participants had withdrawn from participating in the UK Biobank since the GWAS were published and were therefore not included in any individual-level analyses in our study. Participants were categorised as either having MDD with reported trauma or MDD without reported trauma. Subsequently, participants were categorised as having either recurrent or single-episode MDD. There is some degree of overlap between the categories (Fig. 1).

### Posttraumatic stress disorder

PTSD phenotypes can reflect sample characteristics and data collection methods. We used three sets of PTSD GWAS summary statistics to examine whether our findings were consistent across differing PTSD phenotypes. First, we calculated genetic correlations using summary statistics from a GWAS of probable PTSD in the UK Biobank which was based on self-reported answers in the Mental Health Questionnaire (MHQ) (UKB-PTSD). Secondly, we used summary statistics a GWAS of 59 mainly clinical PTSD samples from the PGC (PGC1.5-PTSD) (Nievergelt et al., 2019). Finally, we used summary statistics from a PTSD GWAS based on electronic health records of US veterans by the Million Veteran Program (MVP-PTSD) (Stein et al., 2021). We generated PRS using this MVP-PTSD phenotype.

The number of cases and controls and the SNP-based heritability (liability scale) of each GWAS can be found in Table 1. All summary statistics were produced from GWAS on individuals of European ancestries. Details of the contributing studies and phenotype definitions can be found in online Supplementary Methods.

### Reported trauma in individuals with recurrent and single-episode major depressive disorder

We tested the assumption behind our second hypothesis: we expected the rates of trauma exposure to be higher among individuals who have experienced recurrent compared to single-episode MDD. The UK Biobank participants were categorised as having experienced either recurrent or single-episode MDD, as defined by Coleman et al. (2019). Seven traumatic life events were included in the Coleman et al. (2020) definition of ‘reported trauma exposure’ due to them having a >2.5 odds ratio (OR) with MDD (Table 3). We performed χ^2^ tests in *R* to establish whether there were differences in trauma reporting rates between individuals with recurrent and single-episode MDD. χ^2^ statistics were considered significant if they reached or surpassed the Bonferroni-corrected *α* (0.05/7 = 0.007; to correct for the seven tests performed).

Throughout this paper, any mention of trauma exposure in UK Biobank participants refers specifically to retrospective, self-reported traumatic events due to the nature of data collection via the online MHQ. The events being reported may have occurred before, after or concurrently with MDD episodes.

### Genetic correlations

GWAS summary statistics were used to calculate genetic correlations based on single nucleotide polymorphisms (SNP-based *r*_g_) using High Definition Likelihood (HDL) and the 1 029 876 quality-controlled UK Biobank imputed HapMap3 SNPs reference panel. This reference panel is based on genotypes in the UK Biobank, which were imputed to HRC and UK10K + 1000 Genomes (Ning, Pawitan, & Shen, 2020).

First, we calculated genetic correlations between PTSD and (1) MDD with reported trauma, (2) MDD without reported trauma, (3) recurrent MDD and (4) single-episode MDD within UKB-PTSD. We repeated these genetic correlations using PGC1.5-PTSD and MVP-PTSD. Genetic correlations were tested for a significant difference from 0 (default in HDL) and from 1 (in Microsoft Excel, converting *r*_g_ to a χ^2^ as [(*r*_g_ − 1)/se]^2^). An explanation of HDL can be found in Ning et al. (2020). Genetic correlations were considered significantly different to 0 or to 1 if they surpassed the Bonferroni-corrected *α* (0.05/4 = 0.0125; to correct for the four tests per PTSD phenotype).

To test the significance of the differences between the genetic correlations, we performed a block-jackknife, which uses resampling to recalculate standard errors for the differences between two *r*_g_ estimates. We compared *r*_g_ estimates in a pairwise fashion, where each correlation pair was compared with all other correlation pairs within its group. This resulted in six different block-jackknife tests per PTSD phenotype. Differences between genetic correlations were considered statistically significant if they surpassed the Bonferroni-corrected *α* (0.05/6 = 0.0083; to correct for the six tests).

To maximise power, the PGC combined PGC1.5-PTSD and UKB-PTSD. We repeated these analyses with these summary statistics (PGC2-PTSD). Results can be found in online Supplementary Tables S1 and S5. Online Supplementary Methods contain further details of the HDL analysis, including the percentage overlap between the summary statistics and the HapMap3 reference panel. Online Supplementary Tables S3 and S4 contain the genetic correlations between the PTSD phenotypes and MDD categories, respectively.

We also ran these analyses using Linkage Disequilibrium Score Regression (LDSC), another command line tool for estimating heritability and genetic correlations from GWAS summary statistics (Bulik-Sullivan et al., 2015). In our study, we favoured HDL for estimating genetic correlations. Unlike LDSC, HDL uses a full likelihood-based method to estimate genetic correlations that fully accounts for linkage disequilibrium (LD) across the genome. When compared to LDSC, HDL reduces the variance of the genetic correlation by approximately 60% (Ning et al., 2020). Consequently, HDL is better powered to detect significant differences between correlations, which was a central aim of our study. The LDSC results and an explanation of any differences from HDL are presented in online Supplementary Results.

### Polygenic risk scores

We computed PTSD PRS using PRSice v2.3.1. We controlled for the first six principal components, genotyping batch, assessment centre and current depression severity assessed by the Patient Health Questionnaire 9 (PHQ9). A significant difference in PHQ9 severity was found between the MDD with and without reported trauma group (*W* = 45 762 133, *p* < 2.2 × 10^−16^) and the recurrent and single-episode MDD group (*W* = 70 764 216, *p* < 2.2 × 10^−16^). Medians and IQRs are presented in Table 2. Therefore, PHQ9 severity was included as a covariate to control for negative mood at the time of recall influencing the reporting of traumatic events (Reuben et al., 2016).

PRS were calculated at 11 *p* value thresholds (5 × 10^−8^, 1 × 10^−5^, 1 × 10^−3^, 0.01, 0.05, 0.1, 0.2, 0.3, 0.4, 0.5, 1). Phenotype permutations were used to produce an empirical *p* value for the association at the best-fitting PRS, which accounts for testing at multiple thresholds (Euesden, Lewis, & O’Reilly, 2015). Once the best-fitting PRS had been calculated, we performed logistic regressions to examine whether genetic risk for PTSD showed a greater association with MDD with reported trauma or MDD without reported trauma, and with recurrent or single-episode MDD. The standardised *β* coefficients were converted to OR and 95% confidence intervals were calculated. The full six pairwise comparisons, as in the block-jackknife analysis, were not possible due to overlapping MDD categories (Fig. 1). Therefore, we limit the PRS analysis to two comparisons.

We performed power calculations using the Additive Variance Explained and Number of Genetic Effects Method of Estimation (AVENGEME) programme (Dudbridge, 2013). Details are presented in online Supplementary Methods. MVP-PTSD summary statistics were chosen based on their power and no overlap with the UK Biobank, as overlap between the training and target samples can lead to overfitting. Bonferroni adjustment was used to correct for the two tests, giving a final threshold of *p* < 0.025.

## Results

### Sample characteristics

Table 2 contains descriptive statistics, for age at MHQ, sex and current depression severity (assessed by the PHQ9) for the study sample as a whole and for the four MDD categories.

### Reported trauma in individuals with recurrent and single-episode major depressive disorder

Each of the seven life events comprising the definition of ‘reported trauma exposure’ in Coleman et al. (2020) was significantly more commonly reported by participants who reported recurrent depressive episodes than single-episode MDD (Table 3). Nine further traumatic life events were also assessed by the UK Biobank MHQ. The difference in reporting rates between recurrent and single-episode MDD and the results of the χ^2^ tests can be found in online Supplementary Table S2.

### Genetic correlations

All genetic correlations were significantly different to 0. The genetic correlation between PGC1.5-PTSD and single-episode MDD did not differ significantly from 1, although this is likely due to the large standard errors of the *r*_g_ estimates, reflecting low power. All other genetic correlations were significantly different to 1 (Table 4).

### Differences between genetic correlations

The genetic correlation between PTSD and recurrent MDD was significantly greater than that between PTSD and MDD without reported trauma when using UKB-PTSD and PGC1.5-PTSD (and PGC2-PTSD, which is presented in online Supplementary Results). All other genetic correlations were not significantly different from each other (online Supplementary Table S5). Genetic correlation estimates of PTSD with MDD with reported trauma were consistently higher than those with MDD without reported trauma, albeit not significant (*p* = 0.14–0.65). By contrast, no consistent pattern was observed between PTSD and recurrent v. single-episode MDD (Table 4, online Supplementary Table S5). These results were also observed when using PGC2-PTSD (online Supplementary Tables S1 and S5).

### Polygenic risk scores

In individuals with MDD in the UK Biobank, genetic loading for PTSD was significantly associated with an increased likelihood of reporting trauma [OR 1.04 (95% CI 1.01–1.07), Empirical-*p* = 0.02]. In contrast, those with a higher genetic loading for PTSD were more likely to have experienced a single depressive episode rather than recurrent episodes, but this was not significant [OR 0.97 (95% CI 0.95–0.99), Empirical-*p* = 0.08]. The variance explained by the PRS ranged from 0.03% to 0.06% based on varying the population prevalence of the target phenotype. See online Supplementary Results for details of this analysis, including the number of SNPs in each PRS and Nagelkerke’s *R*^2^ for a range of population prevalences (online Supplementary Table S8).

## Discussion

We investigated whether PTSD and MDD share a genetic component related to being exposed to traumatic events and experiencing internalising symptomatology. We addressed this by measuring the genetic overlap between PTSD and MDD with reported trauma and compared this to the genetic overlap between PTSD and MDD without reported trauma. We aimed to discover whether the genetic variants associated with MDD in individuals reporting trauma were shared with PTSD, which requires trauma exposure by definition. Additionally, we investigated whether genetic risk for PTSD was associated with reporting trauma in UK Biobank participants with MDD. Across all PTSD phenotypes, as hypothesised, genetic correlations with MDD in individuals reporting trauma were greater than genetic correlations with MDD in individuals not reporting trauma. However, the differences were not significant so strong conclusions cannot be drawn from this analysis alone. By contrast, the PRS analysis showed that genetic loading for PTSD in individuals with MDD was associated with a higher likelihood of reporting trauma. This result appears to be robust to recall bias since we controlled for depression severity at the time of reporting.

A potential explanation for this finding involves G × E. Certain individuals may be particularly sensitive to adverse life events due to their inherited genetics, and therefore have a propensity to develop psychopathology. This appears to be the case for PTSD symptoms: experiencing trauma is common, but only a minority of those who experience trauma develop PTSD (Auxéméry, 2012; Duncan, Cooper, & Shen, 2018a; Nievergelt et al., 2019). In our study, the PRS generated from the MVP-PTSD summary statistics capture the risk from common additive genetic variants for a persistent, negative response to traumatic events. Accordingly, the PTSD PRS represent a genetic component of trauma sensitivity in UK Biobank participants with MDD. We found a significant association between the PTSD PRS and reporting trauma in these individuals which suggests that there are genetic variants associated with PTSD that also influence an individual’s sensitivity to trauma in MDD. This can be viewed as a form of G × E in line with the diathesis-stress approach, where the experience of certain life events increases the likelihood of developing psychopathology by activating a genetically driven vulnerability (Colodro-Conde et al., 2018; Meehl, 1962; Monroe & Simons, 1991). Research into G × E between adverse life events and the risk for MDD has previously yielded inconsistent results (Coleman et al., 2020; Colodro-Conde et al., 2018; Mullins et al., 2016; Peyrot et al., 2014). Our study adds to the literature suggesting that genetic risk for PTSD could reflect an underlying dimension of sensitivity to psychologically distressing events. Accordingly, having a higher genetic loading for PTSD may be associated with an increased risk of experiencing internalising symptoms in individuals who have experienced trauma.

The PRS finding is interesting in light of our hypothesis that PTSD would show higher genetic overlap with MDD in individuals reporting trauma compared to MDD in individuals not reporting trauma. Although the genetic correlation analysis yielded no conclusive results, the findings from the PRS analysis provide tentative evidence for an association between the genetics of PTSD and reported trauma in MDD. In the genetic correlation analysis, all PTSD phenotypes showed greater genetic overlap with the MDD with reported trauma phenotype compared to the MDD without reported trauma phenotype. However, the differences between the correlations were not significant so it is difficult to fully answer our research question. However, the lack of significance may be due to the limited power of the GWAS from which the summary statistics originated. Given the PRS results, it is possible that the greater genetic correlation between PTSD and MDD with reported trauma, compared to MDD without reported trauma, might be significant if the MDD summary statistics had been produced from better powered GWAS. The Genetic Links to Anxiety and Depression (GLAD) study, which aims to recruit 40 000 participants, will provide an opportunity to replicate these analyses with sufficient power to understand whether the differences were due to chance.

We note that UK Biobank participants who met the criteria for recurrent MDD reported significantly higher rates of trauma exposure in comparison to individuals who met the criteria for single-episode MDD. This corroborates previous psychiatric research that pinpoints exposure to stressful or traumatic events as a key risk factor for subsequent recurrent MDD (Hovens et al., 2015; Nanni et al., 2012; Otte et al., 2016). We expected PTSD to show a greater genetic correlation with the recurrent MDD phenotype compared to the single-episode MDD phenotype but found no evidence of this in the genetic correlation analysis. Findings from the PRS analysis show that genetic risk for PTSD showed stronger associations with single-episode MDD although the effect was small and not significant.

An interesting finding, which was consistent across UKB-PTSD and PGC1.5-PTSD (and PGC2-PTSD presented in online Supplementary Results), was the significantly higher genetic correlation between PTSD and recurrent MDD compared to PTSD and MDD without reported trauma. This might reflect similarities between PTSD and recurrent MDD. It is known that exposure to trauma, especially in childhood, is related to MDD that is severe and treatment-resistant, as well as recurrent, in later life (Danese, 2020; Nanni et al., 2012). Potentially, in terms of MDD subtypes, MDD without reported trauma may capture participants with milder symptoms, while the recurrent MDD subtype may capture severer symptoms due to the higher reported trauma in this category. Like recurrent MDD, PTSD is a severe psychiatric disorder where full, clinically significant symptoms may present many years after exposure (Kessler et al., 2017). Taking this into consideration, PTSD might share genetic variants associated with symptom severity and persistence with recurrent MDD, which may be shared to a lesser extent with MDD in individuals not reporting trauma. This could explain the significant difference between the genetic correlations. However, if this was the case, we would expect the finding to have replicated with MVP-PTSD. This is because war- and combat-related PTSD tends to be more severe and long-lasting compared to PTSD from other traumas (Kessler et al., 2017). Furthermore, war- and combat-related PTSD has been shown to correlate with heightened symptom severity (Guina, Nahhas, Sutton, & Farnsworth, 2018). As shown in Table 1, at least a quarter of the MVP-PTSD sample had been exposed to combat (Stein et al., 2021).

Unique features of the MVP-PTSD sample may explain why this significant finding did not replicate. First, unlike the UK Biobank and PGC samples (which have a more balanced sex division), MVP-PTSD overrepresents males (94.4%). Previous GWAS findings suggest that PTSD’s heritability differs between men and women (Nievergelt et al., 2019). Secondly, although PTSD is often severe and disabling, it is not a homogeneous disorder (Smith, Summers, Dillon, & Cougle, 2016). It is well known that the nature of trauma(s) can affect subsequent clinical presentation (Kelley, Weathers, McDevitt-Murphy, Eakin, & Flood, 2009). War- and combat-related trauma has been found to be particularly associated with intrusive symptoms and arousal, such as excessive startle and physical reactivity (Guina et al., 2018). It is possible that the type of PTSD measured by the MVP in war veterans differs from that measured in civilians (such as the participants in UKB-PTSD and some participants in the PGC samples), which may alter its genetic sharing with internalising disorders such as MDD.

### Merits and limitations

We were able to use a variety of PTSD definitions and data from the largest PTSD GWAS to date. These samples recruited participants who had experienced different types of trauma, exhibited varying levels of severity and were recruited in distinct ways. To participate in the UK Biobank, individuals visited recruitment centres for a number of hours to undergo physical assessments, provide data and a DNA sample (Sudlow et al., 2015). This level of investment may mean that people who were experiencing severe emotional and functional impairment were unlikely to participate. Contrastingly, the majority of the PGC1.5-PTSD participants were recruited directly from clinical studies of PTSD, using telephone diagnostic interviews and face-to-face clinical assessments (Nievergelt et al., 2019). Consequently, it is reasonable to assume that, on average, the participants comprising this sample report more severe symptoms than individuals drawn from the population without specific ascertainment for mental illness (as is the case with the UK Biobank). In contrast to the UK Biobank and PGC, the MVP sample was limited to US veterans (Stein et al., 2021). The benefit of using varying PTSD phenotypes was that it allowed us to examine whether the extent that trauma sensitivity is shared between MDD and PTSD depends on sample-specific characteristics. We saw that the significantly higher genetic correlation between PTSD and recurrent MDD compared to PTSD and MDD without reported trauma replicated when using PGC1.5-PTSD, suggesting this result is not only applicable to UK Biobank participants with probable PTSD but also to clinically defined PTSD. Likewise, the general pattern of the genetic correlation results was consistent across all PTSD phenotypes.

We note that the MDD phenotypes were defined in UK Biobank participants who show a ‘Volunteer selection bias’ (Fry et al., 2017). This refers to the tendency of research participants to be more health-conscious and have a higher level of social capital than non-participants (Manolio et al., 2012). Therefore, although the UK Biobank offers the opportunity to amalgamate genetic and phenotypic in a large, homogenous, single-population cohort, its demographic features mean the MDD and trauma-related phenotypes cannot fully represent the experiences of other populations.

The interpretation of the results in this study is also affected by the fact that trauma exposure was measured retrospectively. This, and the older age of UK Biobank participants, may lead to inaccurate reporting of events (Colman et al., 2016). Secondly, the lack of temporal information regarding the onset of MDD in relation to traumatic experiences means we cannot infer causality between them. To overcome this, we could have limited the definition of trauma to the three childhood items which would have allowed a more robust measurement of the influence of trauma exposure on the later development of MDD. However, Coleman et al. (2020) reported that limiting the GWAS of MDD with reported trauma to only the events in the Childhood Trauma Screener did not significantly alter the SNP-based heritability of MDD, suggesting that the inclusion of the adulthood events is valid when investigating the relationship between reported trauma and MDD (Coleman et al., 2020). Overall, although this method of measuring trauma exposure is not ideal, it is the only feasible method for collecting large amounts of data required for genomic analyses.

Lastly, our results may not generalise to non-European populations. This limitation, which means the experiences of non-European individuals fail to be accounted for in genetics research, is increasingly being acknowledged. A recent PTSD GWAS from the MVP included individuals of African ancestries (Stein et al., 2021). Sample sizes are currently small but will hopefully grow as the field responds to the need for inclusivity and diversity in its research.

Overall, our findings tentatively point toward the existence of genetic variants which may interact with life events and influence the risk of experiencing internalising symptoms, although replication with better powered GWAS is needed. Our paper makes a step towards understanding the nature of trauma sensitivity in individuals with MDD, whether this has a genetic basis and whether this is shared with PTSD.

## Supplementary Material

Supplementary Material

## Figures and Tables

**Table 1.** Information about the four posttraumatic stress disorder (PTSD) and four major depressive disorder (MDD) genome-wide association study (GWAS) summary statistics, including the original publication, characteristics of the sample, number (*N*) of cases and controls in original GWAS, liability scale SNP-based heritability ($h_{\text{SNP}}^{2}$) and standard error (s.e.) from High Definition Likelihood

Phenotype	Paper containing original GWAS	Sample characteristics	*N* cases	*N* controls	$h_{\text{SNP}}^{2}$ (liability scale)	s.e
UK Biobank PTSD	Nievergelt et al. (2019)	Probable PTSD phenotype was defined in UK Biobank participants based on self-report answers to PTSD Checklist (PCL) 6 (Civilian version) in the Mental Health Questionnaire	10 389	115 799	0.20	0.009
Psychiatric Genomics Consortium 1.5 PTSD	Nievergelt et al. (2019)	Sample comprises 59 studies of PTSD. Most cases were clinically ascertained through telephone or face-to-face interviews	12 823	35 648	0.06	0.011
Million Veteran Program PTSD	Stein et al. (2021)	Sample comprises US veterans. PTSD was algorithmically defined based on electronic health records Confirmed war- and combat exposure: 27.5% No exposure: 29.3% Unknown exposure: 43.1%	36 301	178 107	0.06	0.015
MDD with reported trauma	Coleman et al. (2020)	Phenotype was defined in UK Biobank participants. Sample comprises cases who met criteria for MDD and controls who did not meet criteria for MDD based on answers to the Composite International Diagnostic Interview Short Form (CIDI-SF). Cases and controls reported at least two traumatic life events (Table 3) in the Mental Health Questionnaire	13 393	10 701	0.24	0.017
MDD without reported trauma	Coleman et al. (2020)	Phenotype was defined in UK Biobank participants. Sample comprises cases who met criteria for MDD and controls who did not meet criteria for MDD based on answers to the Composite International Diagnostic Interview Short Form (CIDI-SF). Cases and controls reported no traumatic life events (Table 3) in the Mental Health Questionnaire	9487	39 677	0.15	0.020
Recurrent MDD	Coleman et al. (2019)	Phenotype was defined in UK Biobank participants. Cases met criteria for MDD based on answers to the Composite International Diagnostic Interview Short Form (CIDI-SF) and reported more than one depressive episode in the Mental Health Questionnaire. Controls did not meet criteria for MDD	17 451	63 482	0.22	0.009
Single-episode MDD	Coleman et al. (2019)	Phenotype was defined in UK Biobank participants. Cases met criteria for MDD based on answers to the Composite International Diagnostic Interview Short Form (CIDI-SF) and reported one depressive episode in the Mental Health Questionnaire. Controls did not meet criteria for MDD	12 024	63 482	0.10	0.008

Further details of the phenotypes and how observed scale $h_{\text{SNP}}^{2}$ estimates were converted to the liability scale are presented in online Supplementary Material

**Table 2.** Descriptive statistics for age, sex and current depression severity of the study sample of individuals who met criteria for lifetime major depressive disorder (MDD) in UK Biobank Mental Health Questionnaire (MHQ) respondents

	Sample overall	MDD with reported trauma	MDD without reported trauma	Recurrent MDD	Single-episode MDD
***N***	29 471	13 391	9486	17 449	12 022
Age					
Min	46	47	46	47	46
Max	80	80	80	80	80
Mean	62.36	61.66	63.24	61.83	63.14
Std	7.52	7.43	7.57	7.47	7.53
Sex					
Females (%)	20 323 (69%)	10 248 (77%)	5782 (61%)	12 295 (70%)	8028 (67%)
Males (%)	9148 (31%)	3143 (23%)	3704 (39%)	5154 (30%)	3994 (33%)
PHQ9 severity					
Min	0	0	0	0	0
Max	27	27	27	27	27
Median	4.76	5.82	3.49	5.90	3.12
IQR	6	6	5	7	4

Descriptive statistics and sample size (*N*) are given for the study sample as a whole and for the four MDD categories individually. Age was measured in years and refers to the age when the participant completed the MHQ. Sex was acquired from the central registry at recruitment, but in some cases was updated by the participant. Current depression severity was assessed using the Patient Health Questionnaire 9 (PHQ9). Min refers to minimum value, max refers to maximum value, mean refers to the mean value and std refers to standard deviation. PHQ9 severity was not normally distributed so the median and interquartile range (IQR) are provided instead of the mean and standard deviation

**Table 3.** Difference in reporting rates of traumatic life events between individuals with recurrent and single-episode major depressive disorder (MDD) in UK Biobank Mental Health Questionnaire (MHQ) respondents (*N* = 29 471)

Trauma category	Traumatic event	Endorsement in single-episode MDD (%)	Endorsement in recurrent MDD (%)	χ^2^ statistic	*p* value
Childhood emotional abuse	Felt hated by a family member as a child	2352 (20%)	5238 (30%)	405	**4.41 × 10^−90^**
Childhood emotional neglect	Did not feel loved as a child	3121 (26%)	6702 (39%)	498	**2.71 × 10^−110^**
Childhood sexual abuse	Was sexually molested as a child	1217 (10%)	2690 (16%)	175	**6.46 × 10^−4^**
Adulthood emotional abuse	Was belittled by a partner or ex-partner	3887 (32%)	7590 (44%)	370	**1.57 × 10^−82^**
Adulthood physical abuse	Was physically abused by a partner or ex-partner	2005 (17%)	3987 (23%)	166	**4.34 × 10^−38^**
Adulthood sexual abuse	Was forced to have sex against my will by a partner or ex-partner	890 (7%)	2283 (13%)	240	**3.29 × 10^−54^**
PTSD-related: sexual assault	Ever been a victim of sexual assault	2247 (19%)	4764 (28%)	293	**1.07 × 10^−65^**

Traumatic events include three childhood events, three adulthood events and one posttraumatic stress disorder (PTSD)-related event. Differences were considered significant if they surpassed the Bonferroni-adjusted *α* (*p* < 0.007). Significant *p* values are shown in bold.

**Table 4.** High Definition Likelihood (HDL) genetic correlation estimates (*r*_g_), standard errors (s.e.) and 95% confidence intervals (lower and upper CI) of (1) UK Biobank posttraumatic stress disorder (UKB-PTSD), (2) Psychiatric Genomics Consortium 1.5 PTSD (PGC1.5-PTSD) and (3) Million Veteran Program PTSD (MVP-PTSD) with the four major depressive disorder (MDD) categories

PTSD phenotype	MDD phenotype	*r*_g_	s.e.	Lower CI	Upper CI	*p* (diff 0)	*p* (diff 1)
UKB-PTSD	MDD with reported trauma	0.6040	0.0550	0.4962	0.7118	**4.92 × 10^−28^**	**6.02 × 10^−13^**
UKB-PTSD	MDD without reported trauma	0.4701	0.0742	0.3247	0.6155	**2.43 × 10^−1^**	**9.23 × 10^−13^**
UKB-PTSD	Recurrent MDD	0.7134	0.0481	0.6191	0.8077	**1.03 × 10^−49^**	**2.55 × 10^−9^**
UKB-PTSD	Single-episode MDD	0.6466	0.0691	0.5112	0.7820	**8.35 × 10^−21^**	**3.15 × 10^−7^**
PGC1.5-PTSD	MDD with reported trauma	0.5520	0.0746	0.4058	0.6982	**1.35 × 10^−13^**	**1.91 × 10^−9^**
PGC1.5-PTSD	MDD without reported trauma	0.4841	0.1107	0.2671	0.7011	**1.22 × 10^−5^**	**3.16 × 10^−6^**
PGC1.5-PTSD	Recurrent MDD	0.6937	0.0821	0.5328	0.8546	**2.94 × 10^−17^**	**1.91 × 10^−4^**
PGC1.5-PTSD	Single-episode MDD	0.7560	0.1403	0.4810	1.0310	**7.09 × 10^−8^**	0.08
MVP-PTSD	MDD with reported trauma	0.5397	0.0938	0.3559	0.7235	**8.77 × 10^−9^**	**9.24 × 10^−7^**
MVP-PTSD	MDD without reported trauma	0.4859	0.0871	0.3152	0.6566	**2.41 × 10^−8^**	**3.58 × 10^−9^**
MVP-PTSD	Recurrent MDD	0.5600	0.0532	0.4557	0.6643	**6.57 × 10^−26^**	**1.33 × 10^−16^**
MVP-PTSD	Single-episode MDD	0.6291	0.1113	0.4110	0.8472	**1.59 × 10^−8^**	**8.61 × 10^−4^**

*p* (diff 0) refers to *p* value to test whether the *r*_g_ differs from 0. *p* (diff 1) refers to *p* value to test whether the *r*_g_ differs from 1. Genetic correlations were considered significant if they surpassed the Bonferroni-adjusted *α* (*p* < 0.0125). Significant *p* values are shown in bold.

## Appendix

### List of consortium authors.

Adam X. Maihofer, Caroline M. Nievergelt, Dewlen G. Baker, Victoria B. Risborough, Joseph R. Calabrese, Sandro Galea, Dan J. Stein, Nastassja Koen, Shareefa Dalvie, Allison E. Aiello, Andrea L. Roberts, Koenen K. C., Nadia Solovieff, Henry R. Kranzler, Hongyu Zhao, Joel Gelernter, Lindsay A. Farrer, Eric Otto Johnson, John P. Rice, Laura J. Bierut, Nancy L. Saccone, Alexander McFarlane, David Forbes, Derrick Silove, Meaghan O’Donnell, Richard A. Bryant, Miranda van Hooff, Scott R. Sponheim, Seth G. Disner, Robert H. Pietrzak, Chia-Yen Chen, Jordan W. Smoller, Murray B. Stein, Robert J. Ursano, Ronald C. Kessler, Angela G. Junglen, Douglas L. Delahanty, Ananda B. Amstadter, Christina M. Sheerin, Ken Ruggiero, Katie A. McLaughlin, Matthew Peverill, Caldas-de-Almeida JM, S. Bryn Austin, Bizu Gelaye, Michelle A. Williams, Sixto E. Sanchez, Carol E. Franz, Matthew S. Panizzon, Michael J. Lyons, William S. Kremen, Ole A. Andreassen, Anders M. Dale, Bart P. F. Rutten, Christiaan Vinkers, Dick Schijven, Elbert Geuze, Eric Vermetten, Jurjen J. Luykx, Marco P. Boks, Allison E. Ashley-Koch, Jean C. Beckham, Melanie E. Garrett, Michael A. Hauser, Michelle F. Dennis, Nathan A. Kimbrel, Xue- Jun Qin, Karen-Inge Karstoft, Soren B. Andersen, Anders D. Borglum, David Michael Hougaard, Jonas Bybjerg-Grauholm, Laramie E. Duncan, Marie Bμkvad-Hansen, Merete Nordentoft, Ole Mors, P. B. Mortensen, Thomas Werge, Wesley K. Thompson, Yunpeng Wang, Andrew C. Heath, Elliot C. Nelson, Nicholas G Martin, Scott D. Gordon, Erika J. Wolf, Mark W. Logue, Mark W. Miller, Regina E. McGlinchey, William Milberg, Christopher R. Erbes, Melissa A. Polusny, Paul A. Arbisi, Alan L. Peterson, Douglas E. Williamson, Keith A. Young, Maurer D., Alex O. Rothbaum, Lori A. Zoellner, Matig R. Mavissakalian, Norah C. Feeny, P. Roy-Byrne, Barbara O. Rothbaum, Jessica Maples-Keller, Alaptagin Khan, Jonathan D. Wolff, Lauren A. M. Lebois, Martin H. Teicher, Milissa L. Kaufman, Sherry Winternitz, Elizabeth A. Bolger, Antonia V. Seligowski, Holly K. Orcutt, Alexandra Evans, Bozo Lugonja, Catrin Lewis, Ian Jones, Jonathan I. Bisson, Samuel A. McLean, Sarah D. Linnstaedt, Edward S. Peters, Ariane Rung, Edward Trapido, Megan Brashear, Adriana Lori, Alicia K. Smith, Bekh Bradley, Charles Gillespie, Guia Guffanti, Kerry J. Ressler, Lynn M. Almli, Tanja Jovanovic, Angela C. Bustamante, Monica Uddin, Leigh Luella van den Heuvel, S. Seedat, S. M. J Hemmings, Aferdita Goci Uka, Alma Dzubur Kulenovic, Christiane Wolf, Dragan Babic, Esmina Avdibegovic, Jurgen Deckert, Katharina Domschke, Miro Jakovljevic, Charles Marmar, Janine D. Flory, Marti Jett, Rachel Yehuda, Rasha Hammamieh, Anthony P. King, Israel Liberzon, Julia S. Seng, Charles Phillip Morris, Divya Mehta, Joanne Voisey, Lawford B. R., Ross McD. Young, Sarah McLeay, Nikolaos P. Daskalakis, Sonya B. Norman, Zhewu Wang, Gerome Breen, Jonathan R.I. Coleman, Torsten Klengel, Katy Torres, Karmel W. Choi, Daniel F Levey, Renato Polimanti, Elizabeth G. Atkinson, Alicia R. Martin, Benjamin M. Neale, Mark J. Daly, Stephan Ripke, Andrew Ratanatharathorn, Allison C. Provost, Magali Haas, Jennifer S. Stevens, Rajendra A. Morey, Supriya Harnal, Preben B. Mortensen, Soraya Seedat, Jennifer A. Sumner.
